# Supplementary material for: An adaptive, asymptomatic SARS-CoV-2 workforce screening program providing real-time, actionable monitoring of the COVID-19 pandemic
Source: PLoS One. 2022 May 6;17(5):e0268237. doi: 10.1371/journal.pone.0268237 (PMC9075622; doi:10.1371/journal.pone.0268237)
Supplement: Attachment — Submitted filename: An Adaptive, Asymptomatic SARS-CoV-2 Workforce Screening Program.docx [file pone.0268237.s001.docx]

**An Adaptive, Asymptomatic SARS-CoV-2 Workforce Screening Program**

**Editorial comments**

- Lines 65-66: Local and regional containment strategies are important to allow safe reopening of the country [10]. Which country? Or do you mean countries?
- Line 66: …. …regional containment strategies are important to allow safe reopening of the country [10]. Line 66: SARCoV-2 has been described in fully vaccinated individuals. There is a jump from one idea to the next. There is a need for connector between these two sentences.
- Line 70: SARS-CoV-2 PCR-based.
- Line 71: turnaround times aimed at asymptomatic
- Line 154: ….CoV 2 PCR testp positive….
- Line 164: “One in two”, may be half?

**General comments**

- According to Line 7, the aim of this paper is to “describe a sustained and adaptable SARS-CoV-2 PCR based testing program with rapid turnaround aimed at asymptomatic workforce members, as part of a multilayered institutional COVID-19 mitigation strategy at a pediatric specialty hospital that treats immunocompromised patients”. This is not a sufficient aim for a paper. For the paper to be impactful, it should not only describe but also critically assess or analyze the program.
- The methods section, as currently presented, does not describe the methods used in the paper or study. Rather, the section describes the **Asymptomatic SARS-CoV-2 Workforce Screening Program** as part of a wider Covid-19 management program. Therefore, the section should correctly be given the heading Program description. On the contrary, the results section presents various statistical measures as part of the evaluation of the program. The methods used in the calculation of the statistical parameters and the overall analysis or evaluation of the program should be presented in the methods section. How were asymptomatic workers identified, how were vaccinated employees identified? How was sequencing conducted? How was follow-up conducted?
- The paper has some time-to-event data in form of “median time from second vaccine dose to positive SARS CoV-2 PCR test. How was this assessed? Was a survival analysis conducted
- Why are demographic data only provided for vaccinated employees?
- Is it possible to tabulate some of the information provided in the results section?
- In the results section, vaccination results are presented by name or type of vaccine. Is it not necessary to discuss the results by type of vaccine.
