# Supplementary material for: An adaptive, asymptomatic SARS-CoV-2 workforce screening program providing real-time, actionable monitoring of the COVID-19 pandemic
Source: PLoS One. 2022 May 6;17(5):e0268237. doi: 10.1371/journal.pone.0268237 (PMC9075622; doi:10.1371/journal.pone.0268237)
Supplement: Attachment — Submitted filename: Response to reviewers.docx [file pone.0268237.s002.docx]

April 9, 2022

Re: Response to reviewer comments received March 21st , 2022, for manuscript PONE-D-21-19497: “An Adaptive, Asymptomatic SARS-CoV-2 Workforce Screening Program Providing Real-Time, Actionable Monitoring of the COVID-19 Pandemic.”

We thank the reviewers and editors for their time and insightful comments (noted in italics). Please find below a point-by-point response.

**Editorial comments**

*Lines 65-66: Local and regional containment strategies are important to allow safe reopening of the country [10]. Which country? Or do you mean countries?*

Response: We meant countries. This has been corrected.

*Line 66: …. …regional containment strategies are important to allow safe reopening of the country [10]. Line 66: SARCoV-2 has been described in fully vaccinated individuals. There is a jump from one idea to the next. There is a need for connector between these two sentences.*

Response: This section has been edited as follows:

“Testing played a significant role in pandemic containment during the height of the pandemic, through phases of sporadic cases or clusters, even as overall transmission has declined, especially in areas with uneven geographic vaccine coverage [9]. Specifically, the establishment of asymptomatic testing programs to mitigate COVID-19 have been used in both healthcare and not healthcare settings. While SARS-CoV-2 has been described in fully vaccinated individuals [2, 10-13], the role and value of asymptomatic testing in this group is not well described, although, the US CDC has recommended halting this workplace practice [14]. In addition to widespread available testing, local and regional containment strategies are important to allow safe reopening of countries [15].”

*Line 70: SARS-CoV-2 PCR-based.*

Response: This has been corrected.

*Line 71: turnaround times aimed at asymptomatic*

Response: This has been corrected.

*Line 154: ….CoV 2 PCR testp positive….*

Response: This has been corrected.

*Line 164: “One in two”, may be half?*

Response: This has been modified.

**General comments**

*According to Line 7, the aim of this paper is to “describe a sustained and adaptable SARS-CoV-2 PCR based testing program with rapid turnaround aimed at asymptomatic workforce members, as part of a multilayered institutional COVID-19 mitigation strategy at a pediatric specialty hospital that treats immunocompromised patients”. This is not a sufficient aim for a paper. For the paper to be impactful, it should not only describe but also critically assess or analyze the program.*

Response: We have more than described the program. We have shown the feasibility of an asymptomatic SARS-CoV-2 workforce screening program, its impact on detection of asymptomatic infections and resultant potential days of transmission averted underscoring its values as an important component of a multifaceted risk mitigation strategy and its iterative adaption based on the pulse of the pandemic. The last paragraph of the introduction now reads:

“We describe a sustained and adaptable SARS-CoV-2 PCR-based screening program with rapid turnaround times, as part of a multilayered institutional COVID-19 mitigation strategy at a pediatric specialty hospital that treats immunocompromised patients and characterize the course of asymptomatic infections. We show the impact of the program in terms of number of asymptomatic infections detected and potential days of transmission, averted. This program spanned the initiation of a vaccination campaign allowing for the detection of SARS-CoV-2 in 16 asymptomatic, fully vaccinated employees and demonstrates a robust and flexible testing paradigm as a critical component of a multifaceted approach to disease control that can be deployed in future potential outbreaks.”

*The methods section, as currently presented, does not describe the methods used in the paper or study. Rather, the section describes the* ***Asymptomatic SARS-CoV-2 Workforce Screening Program*** *as part of a wider Covid-19 management program. Therefore, the section should correctly be given the heading Program description. On the contrary, the results section presents various statistical measures as part of the evaluation of the program. The methods used in the calculation of the statistical parameters and the overall analysis or evaluation of the program should be presented in the methods section.*

Response: A methods section has been included with this information and the content describing the program was moved to a section titled “Program description” as suggested by the reviewer.

*How were asymptomatic workers identified, how were vaccinated employees identified?*

Response: Initially, we had trained screeners asking for symptoms upon employees entering the restricted access points to campus. Eventually, an app was developed, and most employees adopted the use of the app to self-screen for symptoms daily. In addition, Occupational Health would ask presence or absence of symptoms in those who tested positive for SARS-CoV-2.

*How was sequencing conducted?*

Response: A methods section has been included with information related to SARS-CoV-2 PCR testing and sequencing.

*How was follow-up conducted?*

Response: The following sentence was added in the Program description:

“Follow up of all employees testing positive for SARS-CoV-2 was performed through weekly phone calls during the isolation period until employee met CDC criteria to return to work.”

*The paper has some time-to-event data in form of “median time from second vaccine dose to positive SARS CoV-2 PCR test. How was this assessed? Was a survival analysis conducted?*

Response: A formal survival analysis was not done given the relatively small number of events. Data shown is using descriptive statistics.

*Why are demographic data only provided for vaccinated employees?*

Response: The following table has been added including information on unvaccinated employees.

|  | Asymptomatic screening  N=414 | | Symptomatic/ Post exposure testing*  N=406 | |
| --- | --- | --- | --- | --- |
|  | Unvaccinated  N=398 | Vaccinated  N=16 | Unvaccinated  N=401 | Vaccinated  N=5 |
| Age (median, range) | 41.5 (18.75-72.26) | 55.74 (25.51- 65.62) | 40.39 (18.06-71.53) | 43.33 (28.65 ~ 53.46) |
| Gender  Female  Male |  | | | |
|  | 242 (60.8%) | 8 (50%) | 283 (70.57%) | 4 (80%) |
|  | 156 (39.2%) | 8 (50%) | 118 (29.43%) | 1 (20%) |
| Race  Black  White  Asian  Hispanic  Others |  | | | |
|  | 189 (47.73%) | 4 (25%) | 104 (26.53%) | 1 (20%) |
|  | 162 (40.91%) | 12 (75%) | 243 (61.99%) | 3 (60%) |
|  | 16 (4.04%) | 0 | 10 (2.55%) | 0 |
|  | 17 (4.29%) | 0 | 24 (6.12%) | 0 |
|  | 12 (3.03%) | 0 | 11 (2.81%) | 1 (20%) |
| Days from second vaccine dose (median, range) | NA | 53 (14-94) | NA | 78 (63-92) |
| Type of Vaccine  Pfizer-BioNTech  Moderna |  | | | |
|  | NA | 15 (93.75%) | NA | 5 (100%) |
|  | NA | 1 (6.25%) | NA | 0 |

*Includes testing due to onset of symptoms consistent with COVID-19 or after exposure to someone with COVID-19

*Is it possible to tabulate some of the information provided in the results section?*

Response: A table has been added with the requested demographic information.

*In the results section, vaccination results are presented by name or type of vaccine. Is it not necessary to discuss the results by type of vaccine?*

Response: We appreciate reviewer’s comment. All SARS-CoV-2 cases in fully vaccinated employees described in the manuscript were among employees who received an mRNA vaccine, with only one receiving Moderna. The main reason is that Pfizer-BioNTech was the vaccine that was widely available to our institution and does not reflect any differences related to vaccine effectiveness.
